# 1586. Phase 3/3b Experience with Long-Acting Cabotegravir and Rilpivirine: Efficacy and Safety Outcomes Through Week 96 by Race

**DOI:** 10.1093/ofid/ofac492.109

**Published:** 2022-12-15

**Authors:** Parul Patel, Emilie Elliot, Ronald D’Amico, Louise Garside, Conor Smith, Jeremy Roberts, Joseph W Polli, Moti Ramgopal, Princy N Kumar, Olayemi Osiyemi, Bryan Baugh, Jean A van Wyk

**Affiliations:** ViiV Healthcare, Durham, North Carolina; ViiV Healthcare, Durham, North Carolina; ViiV Healthcare, Durham, North Carolina; PHASTAR, Macclesfield, England, United Kingdom; Parexel International, Sheffield, England, United Kingdom; GlaxoSmithKline, Mississauga, Ontario, Canada; ViiV Healthcare, Durham, North Carolina; Midway Immunology and Research Center, Fort Pierce, FL; Georgetown University Medical Center, Washington, DC, USA, Washington, District of Columbia; Triple O Research Institute, West Palm Beach, Florida; Janssen Research & Development LLC, Raritan, New Jersey; ViiV Healthcare Limited, Brentford, England, United Kingdom

## Abstract

**Background:**

Long-acting cabotegravir + rilpivirine (CAB + RPV LA) administered monthly or every 2 months is the first and only complete LA regimen recommended by treatment guidelines for the maintenance of HIV-1 virologic suppression. This *post hoc* analysis summarizes pooled outcomes for participants in the FLAIR and ATLAS-2M global Phase 3/3b studies through Week (W) 96 by race.

**Methods:**

Data from participants randomized to receive CAB + RPV LA every 4 weeks (Q4W) or 8 weeks (Q8W) in the FLAIR and ATLAS-2M studies were pooled and stratified by race (Black or African American, White, Asian, Other race). Key efficacy endpoints were the proportion of participants with plasma HIV-1 RNA ≥50 copies/mL and HIV-1 RNA < 50 copies/mL at W96 (FDA Snapshot). Incidence of confirmed virologic failure (CVF; two consecutive HIV-1 RNA measurements of ≥200 copies/mL), safety, and treatment satisfaction (HIV Treatment Satisfaction Questionnaire status version [HIVTSQs]) were also assessed.

**Results:**

937 participants received CAB + RPV LA (Q8W, n=327; Q4W, n=610). Overall, 76% (n=711) of participants were White, 16% (n=149) were Black or African American, 4% (n=41) were Asian, and 4% (n=36) were Other race. The median age (range) was 39 years (19–83), and 23% (n=211) were female (sex at birth) (Table 1). At W96, rates of virologic non-response (HIV-1 RNA ≥50 copies/mL) and suppression (HIV-1 RNA < 50 copies/mL) with CAB + RPV LA ranged 0–3% and 83–94%, respectively, across races (Table 2). Rates of CVF ranged 0–2% across races. Excluding injection site reactions (ISRs), drug-related Grade ≥3 adverse events (AEs) occurred in 2% (n=17/937) of participants, ranging 0–3% across races. Drug-related serious AEs occurred in 5 (< 1%) participants (White, < 1% [n=4]; Other race, 3% [n=1]). Overall, 99% (n=8357/8453) of ISR events were Grade 1 or 2; 2% (n=20) of participants withdrew for injection-related reasons. Mean changes in HIVTSQs total scores increased from baseline to W96 across all races, ranging 0.9–3.0.

**Conclusion:**

CAB + RPV LA demonstrated high efficacy and was well tolerated, with increases in participant satisfaction reported from baseline, irrespective of race. These data support CAB + RPV LA as a complete regimen for the maintenance of virologic suppression in individuals with HIV-1.

**Disclosures:**

**Parul Patel, MSW**, GlaxoSmithKline: Stocks/Bonds|ViiV Healthcare Ltd: Employee **Emilie Elliot, MD**, ViiV Healthcare Ltd: Employee **Ronald D’Amico, D.O., MSc**, GlaxoSmithKline: Stocks/Bonds|ViiV Healthcare Ltd: Employee **Louise Garside, PhD**, GlaxoSmithKline: Stocks/Bonds **Jeremy Roberts, MSc**, GlaxoSmithKline: Employee|GlaxoSmithKline: Stocks/Bonds **Joseph W. Polli, PhD**, GlaxoSmithKline: Stocks/Bonds|ViiV Healthcare Ltd: Employee **Moti Ramgopal, MD, FACP, FIDSA**, AbbVie: Grant/Research Support|Gilead Sciences Inc.: Advisor/Consultant|Gilead Sciences Inc.: Grant/Research Support|Gilead Sciences Inc.: Honoraria|Gilead Sciences Inc.: Stocks/Bonds|GlaxoSmithKline: Advisor/Consultant|GlaxoSmithKline: Grant/Research Support|GlaxoSmithKline: Honoraria|GlaxoSmithKline: Stocks/Bonds|Janssen Research & Development LLC: Advisor/Consultant|Janssen Research & Development LLC: Grant/Research Support|Janssen Research & Development LLC: Honoraria|Janssen Research & Development LLC: Stocks/Bonds|Merck: Advisor/Consultant|Merck: Grant/Research Support|Merck: Honoraria|Merck: Stocks/Bonds|Shionogi: Grant/Research Support|ViiV: Advisor/Consultant|ViiV: Grant/Research Support **Princy N. Kumar, MD**, American Gene Technologies: Grant/Research Support|BioHaven: Grant/Research Support|Eli Lilly: Grant/Research Support|Gilead: Advisor/Consultant|Gilead: Grant/Research Support|Gilead: Stocks/Bonds|GSK: Grant/Research Support|GSK: Stocks/Bonds|Johnson&Johnson: Advisor/Consultant|Johnson&Johnson: Stocks/Bonds|Merck: Advisor/Consultant|Merck: Grant/Research Support|Merck: Stocks/Bonds|Moderna: Stocks/Bonds|Pfizer: Stocks/Bonds|Regeneron: Grant/Research Support|TheraTechnologies: Advisor/Consultant|ViiV: Advisor/Consultant **Olayemi Osiyemi, MD**, Gilead: Advisor/Consultant|gsk: Advisor/Consultant|viiv: Advisor/Consultant **Bryan Baugh, MD**, Janssen Research & Development LLC: Employee|Janssen Research & Development LLC: Stocks/Bonds **Jean A. van Wyk, MB,ChB; MFPM**, ViiV Healthcare Limited: I am an employee of ViiV Healthcare|ViiV Healthcare Limited: Stocks/Bonds.

